# Fabrication of Multscale Fractal-Like Structures by Controlling Fluid Interface Instability

**DOI:** 10.1038/srep37187

**Published:** 2016-11-16

**Authors:** Tanveer ul Islam, Prasanna S. Gandhi

**Affiliations:** 1 Suman Mashruwala Advanced Microengineering Laboratory, Department of Mechanical Engineering, Indian Institute of Technology Bombay, 400076, Powai, Mumbai-India

## Abstract

Nature, in quest for the best designs has shaped its vital systems into fractal geometries. Effectual way of spontaneous fabrication of scalable, ordered fractal-like structures by controlling Saffman-Taylor instability in a lifted Hele-Shaw cell is deployed here. In lifted Hele-Shaw cell uncontrolled penetration of low-viscosity fluid into its high-viscosity counterpart is known to develop irregular, non-repeatable, normally short-lived, branched patterns. We propose and characterize experimentally anisotropies in a form of spatially distributed pits on the cell plates to control initiation and further penetration of non-splitting fingers. The proposed control over shielding mechanism yields recipes for fabrication of families of ordered fractal-like patterns of multiple generations. As an example, we demonstrate and characterize fabrication of a Cayley tree fractal-like pattern. The patterns, in addition, are retained permanently by employing UV/thermally curable fluids. The proposed technique thus establishes solid foundation for bio-mimicking natural structures spanning multiple-scales for scientific and engineering use.

Multi-scale fractal structures are found abundantly in nature: some examples are leaf veins, blood vessels, respiratory system, and alike. Several attempts of bio mimicking these structures confirm their effectiveness in enhancing the performance of the application. For example, heat and mass transfer^1^,^2^,^3^, capillary pump^4^, synthetic leaf^5^, passive micromixers^6^, non-transparent solar electrodes^7^, and so on. Effective, scalable method of lithography-less fabrication of such multi-scale, ordered fractal-like structures by controlling fluid interface instability in a Hele-Shaw cell is presented here.

Penetration of a low viscosity fluid into high viscosity counterpart generates a morphologically unstable interface known as Saffman-Taylor instability^8^, which leads to formation of fingering patterns. One of the methods for realizing such an unstable interface is lifted Hele-Shaw cell^9^. A lifted Hele-Shaw cell is an arrangement of two plates, compressing a thin layer of highly viscous fluid between them. An unstable interface with a low viscous fluid (normally air) is created by allowing it to penetrate radially inwards by lifting the plates apart. A variant of Saffman-Taylor instability^10^,^11^, in this case, results in the formation of branched (fractal-like) structures of viscous fluid on both the cell plate surfaces as a mirror image of each other.

Comparatively a limited number of experiments reported for lifted version of Hele-Shaw cell have dealt with topics such as: permanent fractal-like structures produced in non-Newtonian paints^12^,^13^, competition between the penetrating air fingers (shielding mechanism)^14^,^15^,^16^, stretching of yield stress fluid in the cell^17^,^18^, and fingering instability in adhesives^19^. Streaks (branches) of fluid left behind by the penetrating air fingers form a network in which these branches arise at the periphery and join with each other (as against finger splitting in radial Hele-Shaw cell) giving rise to highly branched network. Depending on the type of fluid used, the pattern formed is either short lived or spreads out slowly over time or retains its three dimensional shape permanently. Using anisotropies we propose to exercise control over and retain the fractal-like pattern formed for variety of applications.

Several theoretical and experimental studies report the effect of anisotropies over the pattern morphologies in a linear^20^,^21^,^22^,^23^,^24^,^25^ and radial^26^,^27^,^28^,^29^,^30^,^31^,^32^,^33^ Hele-Shaw cell. Presence of anisotropies in the form of surface textures of various kinds used in these papers have been found to show symmetric growth of limited number of highest generation branches. Similarly experimental and theoretical studies have been reported on use of anisotropies on plates in lifted Hele-Shaw cell^9^,^34^,^35^. Anisotropies used are in form of radial grooves, textures, and lattices. However, these studies show little evidence of control over guaranteed formation of individual finger/branch at given location/s, and/or over number of branches in one generation and/or number of generations.

We propose to use anisotropy on Hele-Shaw plate, especially at the interface of two fluids, to achieve the desired fractal-like pattern (say Cayley tree^36^ with desired coordination number or so) accurately and repeatedly. The proposed anisotropy in the form of small pits is observed to repel branches. Using experimental characterization of effect of pits and their dimensions, we systematically develop a recipe for specifying location and sizes of pits for fabrication of a given pattern. Furthermore we propose to solidify the pattern thus formed by using UV or thermal curing. Although for this development, as an example, we consider Cayley tree pattern with family of fluids, it is easily projected that by using similar concepts, and different kinds of fluids a large class of branched structures spanning multiple scales starting with microscale can be produced for variety of applications.

We report for the first time a unified method for fabrication spanning multiple scales of micro, meso, meter in which the desired structure evolves in a matter of few seconds. The formed structures can be further casted as hollow channels. These structures, having smooth variation in thickness direction (similar to biological structures), can fulfill demanding need for replication of biological structures towards connecting nanoscale to micro and further meso scales for several applications mentioned above and beyond.

## Results

### Control using pits

**Figure 1:**
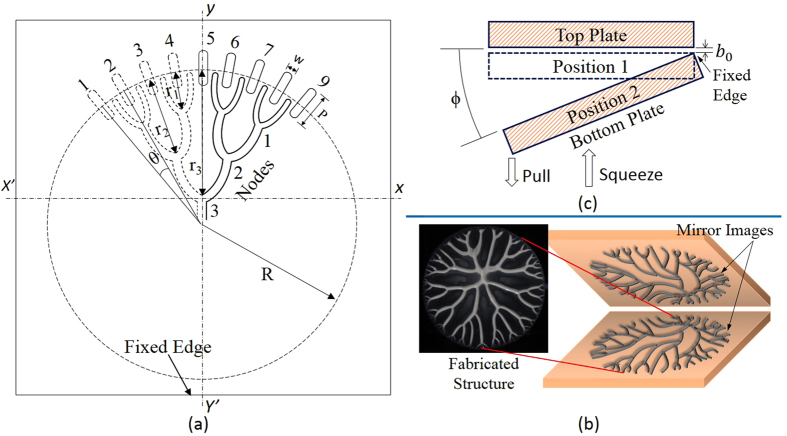
Schematic diagram representation. (**a**) Schematic view of the pits (width *w*) separated by angle *θ*, with reference to squeezed circular fluid film of radius *R*. Shield distances for various generation are *r*_*n*_ (where n is generation number). (**b**) Schematic view of the cell plates showing spontaneous evolution of fractal-like structure (as mirror images of each other) in the absence of control. The figure also shows top view of the actual structure. (**c**) Schematic view of lifted Hele-Shaw cell in which the bottom plate first squeezes the fluid drop to a thickness *b*_0_ followed by its precise angular separation guided by compliant mechanism with remote center motion about opposite edge.

A drop of the high viscous fluid (Bingham-pseudoplastic ceramic suspension in our case) is squeezed between two plates of lifted Hele-Shaw cell shown in Fig. 1(c) followed by angular separation of plates. Precise Angular separation is achieved using compliant “remote center motion” (RCM) mechanism attached to the cell plate. Upon lifting, as observed by several previous works, the fractal-like pattern shown in Fig. 1(b) evolves spontaneously. Normally structures formed in case of Newtonian fluid coalesce into wider branches or finally spread into a single film over an interval of time^15^,^37^. Non-Newtonian fluids which show visco-plastic behavior (fluids with yield stress) produce fractal-like structures that retain their three dimensional branch shape and could later be converted into a solid form by some means. We solidify this pattern permanently using UV curing or thermal curing in our case as against the solvent evaporation used previously^12^,^13^. Thus we propose to use UV/thermal curable resins mixed with ceramic nanoparticles to form a fluid of required visco-plasticity.

The pattern formation process in lifted Hele-Shaw cell involves initiation of air fingers at some random locations on the interface followed by their competition of suppressing each other (termed as shielding mechanism) in a rapidly decreasing area of spread. Hence to generate a desired pattern, it is required to exercise control over these two aspects- initiation and shielding mechanism. Theoretical analysis for complex fluids^19^ indicates that under ideal conditions, the fingers would evolve with wavelength *λ* (= 2*πr*/*N*, N number of fingers), which depends on fluid properties and film geometry. We propose to precisely control finger initiation by introducing anisotropy in form of pits on one of the plates at the interface of compressed fluid blob as shown in Fig. 1(a) marked 1–9. The idea is to seed the locations for initiation of fingers rather than allow them to be initiated randomly as observed experimentally in the absence of pits. The pits act as locations of low resistance for air flow and allow the air finger to penetrate preferentially as the lifting process begins. While designing pit width and their separation the spontaneous wavelength *λ* was taken into consideration. If pit separation is more than around 2.5 times the wavelength, we find that there is susceptibility to generation of additional undesired parasitic fingers. Thus our proposed finger seeding technique is demonstrated experimentally to achieve a high degree of control on initiation beyond the natural wavelength limits.

Although initiation of fingers (and hence placement of the first generation branch tip) is controlled by placement of pits, it is the shielding competition which is responsible for further evolution of the pattern. Shielding mechanism in which penetrating air fingers compete with each other, leave an intricate network of fluid branches on the cell plates. To get precise formation of the desired fractal-like structure, control over propagation (shielding mechanism) of fingers is required. To control propagation of one finger faster than other, we propose to change the depth (*d*) of pits used for initiation. Higher the depth of pit faster is propagation of finger originating from that pit. Thus by controlling the depth of pits 1–9 shown in Fig. 1(a), we can get, for example, a part of Cayley tree having 3 generations/shells with coordination number equal to 3, as explained later in the recipe for fabrication of ordered fractal-like patterns.

With increase in the tip/separation velocities in a linear^38^ or lifted^9^ Hele-Shaw cell, the stability of fingers decreases rapidly leading to an uncontrolled finger splitting. Splitting of fingers forms extra branches which are not desirable for obtaining controlled predefined structures and needs to be restricted by using lower velocities. This limit is obtained experimentally in our case using a single pit at the interface shooting a single big finger into the cell. It was found that for the lowest film thickness (*b*_0_) to radius (*R*) ratio (most propensity for finger splitting), in our case, this limit on angular velocity is 5 × 10^−4^ rad/sec (when *b*_0_ 35 *μm* and R = 16 mm). This limit is maintained in all our experiments to avoid uncontrolled finger splitting.

### Pit depth and initiation time

**Figure 2:**
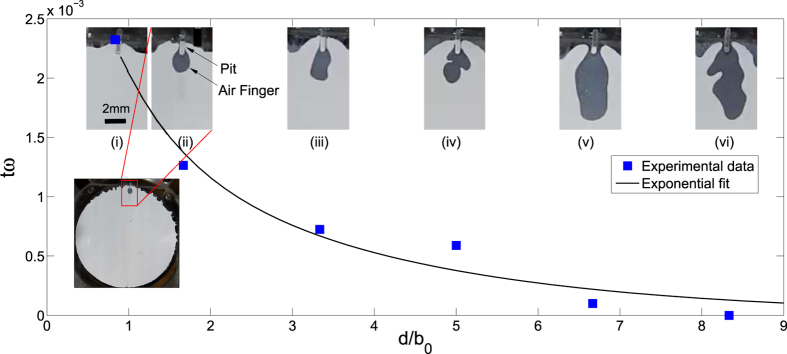
Pit depth and finger penetration length. Graph shows the experimental data obtained by analyzing the emergence of finger from a single pit. The data was obtained by choosing *R* = 18 mm, *b*_0_ = 60^±2^ *μm*, *ω* = 2.5 × 10^−4^ rad/sec, *w* = 500 *μm* and *d* varying from 50–500 *μm*. With *b*_0_ and *ω* constant the graph shows that increase in the pit depth *d* decreases the finger initiation time *t*. Images (*i*)–(*vi*) show the distance travelled by the fingers from pits with increasing *d* in the same time interval as it takes for the finger from pit with minimum *d* to just emerge out of the pit (image (*i*) with *d* = 50 *μm*).

To develop recipe for controlled fabrication, (example of a Cayley tree mentioned earlier in Fig. 1(a)) it is required to characterize experimentally the effect of depth (*d*) of pit on the relative delay (*t*) in finger initiation and hence relative depth of penetration of fingers. Experiments were performed using a digital camera to measure a relative delay in finger initiation under identical conditions except for variation of pit depth. In Fig. 2 as *d*/*b*_0_ → 0, *t* → *t*_*max*_. The delay (*t*) (normalized by angular velocity of separation) goes on decreasing exponentially as the pit depth (normalized by thickness of film (*b*_0_)) increases. For every depth considered, Fig. 2 also shows relative depth of penetration of fingers in the time corresponding to delay with minimum d. Thus with a change in the depth of pit the competition between adjacent fingers can be dictated to produce fractal-like structures with higher generations. From Fig. 2 we observe that higher ratio of depths of adjacent pits will cause stronger shielding and robust control over the structure even in the presence of impurities which may affect the process.

Before proceeding further, following important points regarding consistency and repeatability of these structures are noted: (i) Purpose of pit is to initiate the finger, hence pit width *w* is kept at constant value (500 *μm*). (ii) The natural wavelength (*λ*) for our fluid is estimated to be between 0.5–1.2 (considering uncertainty in some parameters). We deliberately use the pit separation upto a distance 3.75 mm which is way beyond the natural wavelength to demonstrate effectiveness of the proposed anisotropy. (iii) Change in film thickness *b*_0_, as will be shown later, is found to have relatively insignificant effect on structure formations hence constant *b*_0_ = 35^±2^ *μm* is chosen for characterization, and (iv) Angular velocity of separation of lower plate is kept constant at a value of 2.5 × 10^−4^ rad/sec (lower than the limiting value of 5 × 10^−4^ rad/sec mentioned above).

With this preamble about fundamental concepts employed for exercising control over otherwise spontaneous evolution, we now develop algorithm or recipe for fabrication of desired patterns followed by parametric characterization of the patterns formed (control parameters being pit depth “*d*” and spacing “*θ*”).

### Recipe for ordered structure fabrication

With the proposed robust control over finger initiation and shielding mechanism, a Cayley tree pattern with coordination number 3 upto 3 generations (third order structure) as in Fig. 1(a) is considered for fabrication. Although patterns upto 3 generations are considered for fabrication and characterization, the results can be extended for controlled fabrication of similar other patterns (e.g. Cayley tree with higher coordination numbers and higher generations). Fabrication of structures upto fifth generation is demonstrated in the Supplementary Fig. 1. To develop algorithm for setting pit depths, we start with the first generation (branches joining at node 1 at a shielding distance of *r*_1_, Fig. 1(a)). To get the node 1, middle finger originating at 8 needs to be shielded by two side fingers at 7 and 9. Hence the depth of pits at 7 and 9 are kept higher than that at 8. For getting second generation pattern (branches joining first at node 1 and then at node 2 at distance *r*_1_ and *r*_2_ respectively), we need finger originating at 7 to be shielded by those originating at 9 and 5, hence pit at 9 and 5 are kept higher depth than that at 7. Finally the same analogy can be extended to get third and further multiple generation patterns. Hence to get complete pattern upto the third generation, fingers originating at pits 2, 4, 6, and 8 have to be shielded by adjacent fingers and hence the depth of these pits are kept least (50 *μm*). Depths of pit 3, and 7 are to be kept higher than pits 2, 4, 6, and 8 to value of 60 *μm*. Finally fingers originating at pits 1, 5, and 9 have to travel farthest to shield others and hence their depths are kept highest (*d* = 400 *μm* for pit 5 and 500 *μm* for pits 1 and 9). With this algorithm for setting pit depths (*d*), a Cayley tree pattern shown schematically in Fig. 1(a) is obtained. Supplementary Movie 1, shows the evolution of this pattern in our lifted Hele-Shaw cell. The same pattern evolved consistently when the experiment was repeated multiple times. Generalizing the algorithm: for fabrication of a structure of generation N, N + 1 distinct pit depths are required. Under ideal conditions this means no limit over the number of generations. However there will be practical limits and robustness considerations.

**Figure 3:**
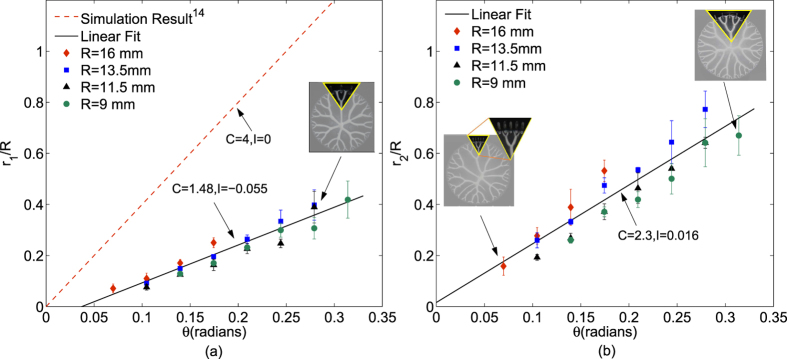
First and second generation shielding distance (**a**) Simulation results show that shielding distance vary linearly against *θ*, with *C* = 4, *I* = 0 where as experimental data shows slope *C* = 1.48 and intercept *I* = −0.055. Data points shown are from the first generation structures fabricated with *R* = 9, 11.5, 13.5, and 16 mm and *θ* varying from 0–0.35 radians while maintaining *b*_0_ = 35 *μm*. (**b**)Shielding distance *r*_2_ again shows a linearly increasing trend with *C* = 2.3 and *I* = 0.016. Inset in the figure shows the structure fabricated as per the parameters indicated. Pictorial graph showing *r*_1_/*R* and *r*_2_/*R* variation against *θ*, for first and second generation structures is shown in Supplementary Fig. 2.

Further, characterization of distances *r*_1_, *r*_2_, and *r*_3_ is performed with respect to geometric variations in position of pits to gauge effectiveness of the proposed control with increasing angle *θ*. Simulations^14^ for only *r*_1_ is carried out by considering a sector of a lifted Hele-Shaw cell with just three fingers propagating towards the centre. Sector geometry in the case of constant gap Hele-Shaw cell has only one finger penetrating at the center followed by its tip widening or ultimately tip-splitting^39^,^40^,^41^,^42^. Theoretical analysis^14^ carried out using artificial numerical disturbance for localization of fingering for a simple Newtonian fluid with parallel separation predicts linear variation of *r*_1_ in the following way:


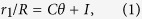


where, *C* is the slope, *I* is the intercept, *R* is the radius of compressed film and *θ* is the angle in radians between two pits. Increase in *θ* is imposed in simulations^14^ by increase in wavelength of numerical artificial perturbation, which increases the distance between the penetrating fingers. Consistent with simulation results for parallel plate separation, our experimental results in Fig. 3(a) for lifted Hele-Shaw cell also showed a linear variation of shielding distance against *θ*, however with significant difference in slope (*C*) and intercept (*I*). Experimentally obtained value of I (= −0.055) is close to simulated value (= 0) however for slope C observation is different. A large difference in the experimental slope C (1.48) and simulated C (4)^14^ can be explained as follows: Numerical perturbation given in the simulation, sets in weak competition between the adjacent fingers as compared to strong competition set in by introduction of depth difference in pits. In addition the azimuthal widening observed in experiments is much more than that predicted by simulation. Moreover, effects of surface tension and wiping energy of the moving fluid boundary are neglected in simulation. As a result the central finger, finally resulting in distance *r*_1_ for node 1, would get shielded earlier in experiments than predicted by simulation. It is found that any depth ratio (<1) between central and adjacent pits produces first order structures with shielding distance *r*_1_ lying within error bands shown in Fig. 3(a).

**Figure 4:**
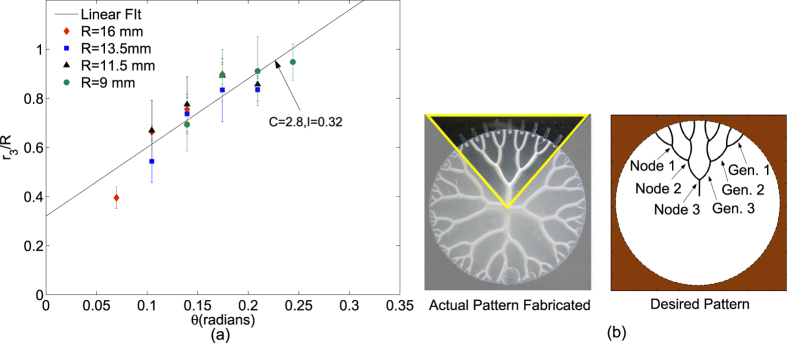
Fabrication of third ordered Cayley tree with coordination number 3. (**a**) Third generation shielding distance *r*_3_ reasonably follows a linear relation with *θ*. Shielding distance *r*_3_ has *C* = 2.8 and *I* = 0.32. Data points shown are from the third order structures fabricated with *R* = 9, 11.5, 13.5, and 16 mm and *θ* varying from 0–0.25 radians while maintaining *b*_0_ = 35^±2^ *μm* for all the experiments. (**b**) Desired and actual pattern of the Cayley tree of coordination number 3 upto third generation.

**Figure 5:**
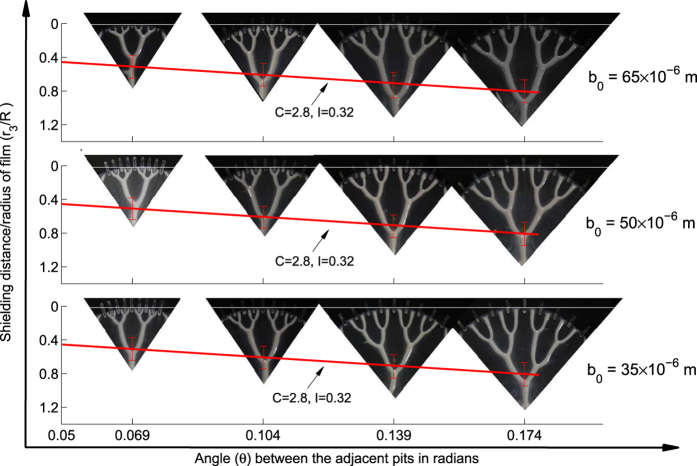
Third order structure image. Pictorial graph shows *r*_3_/*R*, with red line representing the slope against *θ* calculated from data in Fig. 4 along with error bar plotted over it. Structures are fabricated by keeping *R* = 16 *mm* (constant) with three different film thicknesses *b*_0_ = 35 *μm*, *b*_0_ = 50 *μm* and *b*_0_ = 65 *μm*. Slope as well as error bar is shown to remains same in all three cases of *b*_0_. We observe that all the structures fall within the error bars obtained as per Fig. 4(a).

Similar curves for normalized shielding distances *r*_2_/*R* and *r*_3_/*R* in case of Cayley tree with second and third generation structures (with coordination number still 3) are plotted in Figs 3(b) and 4(a) respectively. Desired and actual fabricated third generation structure is shown in Fig. 4(b). These curves also follow linear trend although with different C and I values as mentioned in the respective figures. The results of variation of *r*_1_/*R* and *r*_2_/*R* in these structures, also match the results in Fig. 3(a) and (b) respectively within error bands mentioned, see supplementary Fig. 3. Third generation structures fabricated with increasing *θ* for a given R and for different film thicknesses (*b*_0_) are pictorially shown in Fig. 5. We observe the range of variations in 3rd generation patterns obtained by exercising control over theta and fluid film thickness. Also the change in thickness, although affects the patterns in terms of change of *r*_1_, *r*_2_ and *r*_3_, the effect is relatively insignificant given error bars on them. We observe that the thickness of fluid film has insignificant effect on the slope C considering the error bands around the variation of *r*_3_/*R* with *θ*. Thus the proposed method not only yields the desired pattern but also yields good control over shielding distances in the pattern.

The error bars observed in Figs 3, 4(a) and 5 on the shielding distances *r*_1_, *r*_2_, and *r*_3_ can be explained as follows: Although relative change in the depth of pits gives excellent control over shielding mechanism, fluid impurities (higher size of particle in suspension), micro surface irregularities, and roughness of milled edge of the pit are sources of variation of shielding distances. Shielding distance plotted at a point is average taken over number of samples and error band represents upper and lower values of the distance. Particularly, we observe that the error band on *r*_3_ is higher than that on *r*_1_ and *r*_2_, this is because the shielding in that case has to occur at a farther distance and effect of the above mentioned impurities is amplified as distance increases.

**Figure 6:**
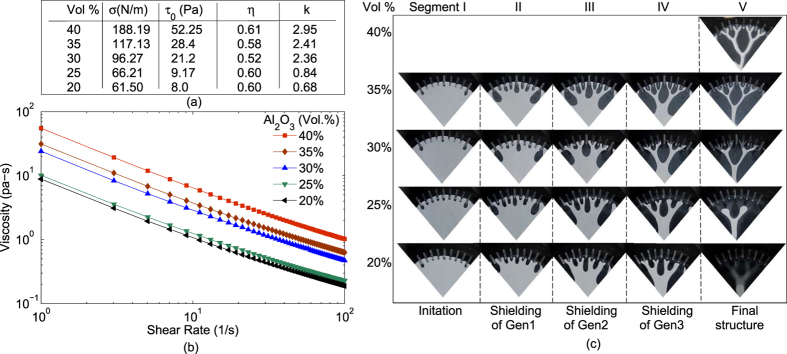
Initiation and shielding with fluid change. (**a**) Surface tension and parameters of Herschel–Bulkley model (*τ* = *τ*_0_ + *kγ*^*η*^) are listed for fluids with particle (*Al*_2_*O*_3_, CT300) loading of 40%, 35%, 30%, 25% and 20% with respect to the volume. (**b**) Figure shows the viscosity change with change in shear stress for fluids with different particle loading. Along with the change in ceramic concentration the surface tension and yield stress of the suspension also decreases. (**c**) Corresponding to the fluids with different rheology the figures (extracted from videos captured during plate separation) show the fingers initiating (segment I) from the pits which then sets in a shielding mechanism such that the fingers of first, second and third generation get terminated, as shown in segment II, segment III and segment IV respectively, in a similar manner in all the cases. Section V shows the final structure left on the cell plates. For a particle loading of 40% only the final structure is shown as the structure is retained permanently and thus finger initiation and termination can easily be interpreted. For fluid with decreasing *γ*, *σ*, and *τ*_0_ the smaller branches start to coalesce and only a part of the structure is retained. Structure is seen to completely recollect into a buckled drop for the least *γ*, *σ*, and *τ*_0_ (20% Vol. fraction). The experiments were done for *b*_0_ = 35 *μm*, *R* = 11.5 *mm* and pit angle (*θ*) = 0.139 *Radian*.

The properties where fluid interactions would show Saffman Taylor instability have been presented in ref. 43. Keeping fundamental principles, of initiation and later propagation of fingers, in pattern generation the same we seed the initiation points and control propagation. Hence it can be seen that the proposed control can be extended to a wider class of fluids. For a few fluids in this class, Fig. 6 shows the evolution of similar Cayley tree pattern in all fluids. Each of the fingers emerging out of pits for all these fluids would be a normal/wide finger because the surface tension is high enough to not allow a pointed/narrow finger^44^,^45^. Thus we can conclude that perturbations of properties of fluid in the class will have relatively insignificant effect on the pattern evolution, thereby indicating strength of the proposed control. We note here that although the shielding of fingers happened in the same order for all fluids, they were retained permanently only for cases where yield stress was high.

## Discussion

It can be concluded that the proposed control, achieved by seeding initiation and regulating shielding of fingers, would produce fractal-like structures upto several generations in a quick, repeated, robust way, and with a reasonable accuracy. As indicated in Supplementary Fig. 4, thickness of these structure increases naturally and smoothly from the narrowest branch to the widest and thus resembles also structures in nature (for example leaf). Several fractal structures appearing in the nature useful for their inherent efficient fluid flow and other structural properties can be fabricated with the least effort by harnessing the proposed control and methodology for interfacial pattern formation. Based on characterization presented in this paper, structures with different branch length ratio (*r*_1_/*r*_2_, *r*_2_/*r*_3_ and so on) can be fabricated resembling a wide diversity of vascular systems in plant and animal kingdoms. Further by changing fluids/suspensions to be used and the pit geometries it is possible to control minimum branch width down to a few microns. Limiting factors towards achieving smaller dimensions for branches are natural impurities and surface roughness, making control due to exceedingly smaller pits weak. On the larger scale side there is virtually no limit as long as the lifting phenomena can be successfully executed. Additional control pits inside the fluid boundary may be needed to be incorporated for better control over the error band in this case. Thus the proposed investigation can have direct implication for bio-mimicking natural structures for several applications including efficient gas exchange (lung/gill mimicking), desalination/distillation (leaf mimicking), blood-vessel flow, etc. and bioinspired multiscale structures for efficient heat exchangers, adsorption, capillary pumping, chemical reactors, mixers, tissue engineering, cell sorting, cell migration, and so on.

## Methods

### Experimental details

Experimental setup depicted in Supplementary Fig. 5 is designed to execute operation of lifted Hele-Shaw cell shown in Fig. 1(c) with a high precision angular motion. Since finger propagation is highly sensitive to even slight shift of axis of rotation of angular motion (especially with lower thicknesses of fluid film), a compliant mechanism with remote center motion (RCM) is employed. Another linear compliant bearing^46^ along with a linear voice coil actuator (LA15-26-000A, from BEI Kimko) is used for compression of fluid in plates and their angular separation guided by RCM mechanism. A linear position encoder (RGH25F, from Renishaw) is used for actuator position feedback and a six-axis load cell (Mini 45 from ATI Industrial Automation) is used for measurement of squeeze and separation forces. A dSPACE DAQ card ds1104 is used for interfacing actuators and sensors and further maintaining appropriate squeeze force and a constant angular velocity of separation via closed loop PI control.

### Fluid Used

A shear thinning fluid showing yeild stress is used to form permanent three dimensional fractal-like structure in the lifted Hele-Shaw cell. The fluid is a photo-resist ceramic suspension prepared from a monomer HDDA (1, 6 Hexanediol diacrylate) by (Sigma Aldrich). Benzoin ethyl ether (BEE) was added as photoinitiator and phosphate ester (PE) as surfactant. Ceramic material selected was alumina (Almatis, CT3000, mean particle size 0.5 microns, refractive index 1.7). Suspension was prepared by adding alumina incrementally in HDDA monomer which was mixed with 4 wt.% of BEE and 2.5 wt.% PE till the solid volume fraction reaches 0.40. Ceria stabilized zirconia balls were used in the ratio of 1:1 with respect to weight of powder to homogenize the suspension in a pot mill. Viscosity of suspension is measured from modular compact rheometer (Anton paar Physica MCR301) by parallel plate method, maintaining a gap of 0.5 mm between them. Wilhelmy plate method used for measuring surface tension measured a value of 188.19 mN/m.

### Design of experiment

A measured quantity of the fluid is placed carefully at the center of the bottom plate and slowly squeezed against the top plate allowing it to spread radially outward to a desired thickness and corresponding radius. A delay of 30 sec is introduced to stabilize the fluid. Separation at a constant angular velocity of 2.5 × 10^−4^ rad/sec is carried out in a closed loop PI control. A camera (18 MP NIKON S9400) records the video at the rate of 30 fps. The frames are processed to determine the delay t in the initiation of finger. ImageJ-software is used to accurately measure distances *r*_1_, *r*_2_, and *r*_3_. Thickness of the branches was recorded using white light interferometry technique (MSA 500, Polytech, Germany).

### Controlling pits

Grooves(pit) are milled in the radial direction as shown in Fig. 1(a) at a radius where the compressed film interface would come to rest before plates detach. Pits are positioned symmetrically on both the sides of *yy*′ axis and on the side opposite to the fixed edge. A pit is meant only to assist in initiating a finger, therefore it is desirable to let the interface to stretch to a minimum length possible over the pit length (P). In all present experiments the distance from the inner edge of pit to the final interface position to which fluid compresses is kept below 1 mm. The pit width (w) is kept at a constant value of 0.5 mm in all experiments, which is less than half the natural initial natural wavelength of an ideal Newtonian fluid of a similar geometry.

## Additional Information

**How to cite this article**: Islam, T. and Gandhi, P. S. Fabrication of Multiscale Fractal-Like Structures by Controlling Fluid Interface Instability. *Sci. Rep.* **6**, 37187; doi: 10.1038/srep37187 (2016).

**Publisher’s note**: Springer Nature remains neutral with regard to jurisdictional claims in published maps and institutional affiliations.

## Supplementary Material

Supplementary Information

Supplementary Movie 1
