# Supplementary material for: Fabrication of Multscale Fractal-Like Structures by Controlling Fluid Interface Instability
Source: Sci Rep. 2016 Nov 16;6:37187. doi: 10.1038/srep37187 (PMC5111118; doi:10.1038/srep37187)
Supplement: Supplementary Information [file srep37187-s1.pdf]

Fabrication of Multiscale Fractal-Like Structure by Controlling Fluid Interface Instability

Tanveer ul Islam[1] **and Prasanna S. Gandhi**[1,*]

[1]Suman Mashruwala Advanced Microengineering Laboratory, Department of Mechanical Engineering, Indian Institute of Technology Bombay, 400076, India
[*]gandhi.iitb@gmail.com

Supplementary Movie. 1. In supplementary movie image gets blur because the top and bottom both plates have structures and bottom plate gets out of focus once lifting begins.

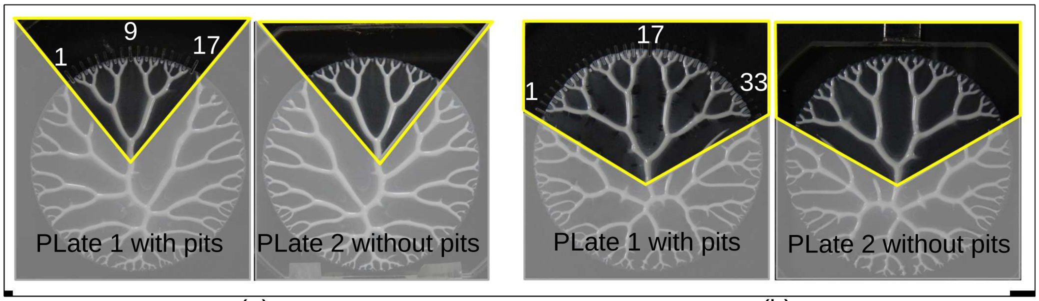

(a) (b)

Supplementary Fig. 1. Figure shows (a) Fourth order structure fabricated with depth of all pits (2,4,6,8,10,12,14,16) producing first generation branches equal to 50 μm, all second generation pits (3,7,11,15) 60 μm, third generation pits (5,13) 150 μm and fourth generation pit (9) equal to 400 μm. (b) Fifth order structure fabricated with all first generation branch producing pits 50 μm deep, second generation pits 60 μm, third generation pits 150 μm, fourth generation pits 300 μm and fifth generation branch producing pit 400 μm deep. Both the structures were produced with $b_0 = 35^{\pm 2}\mu$, $R = 18mm$ and $\theta = 4^0$. Structures are produced as mirror images of each other on both cell plates, one with control pits and one without any pits.

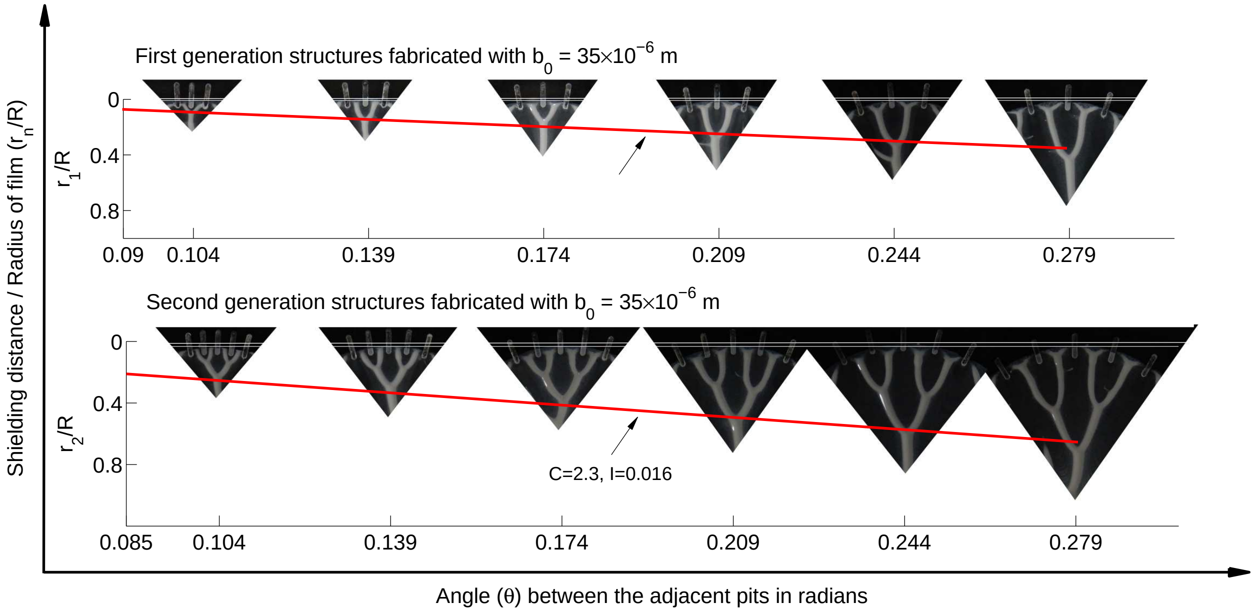

Supplementary Fig. 2. Pictorial graph shows the r_1/R and r_2/R, (for first and second generation structures) with red line representing the slope against θ calculated from data in Fig. 3(a) and Fig. 3(b) respectively. Structures are fabricated by keeping R = 13.5 mm (constant) with film thickness $b_0 = 35\mu m$

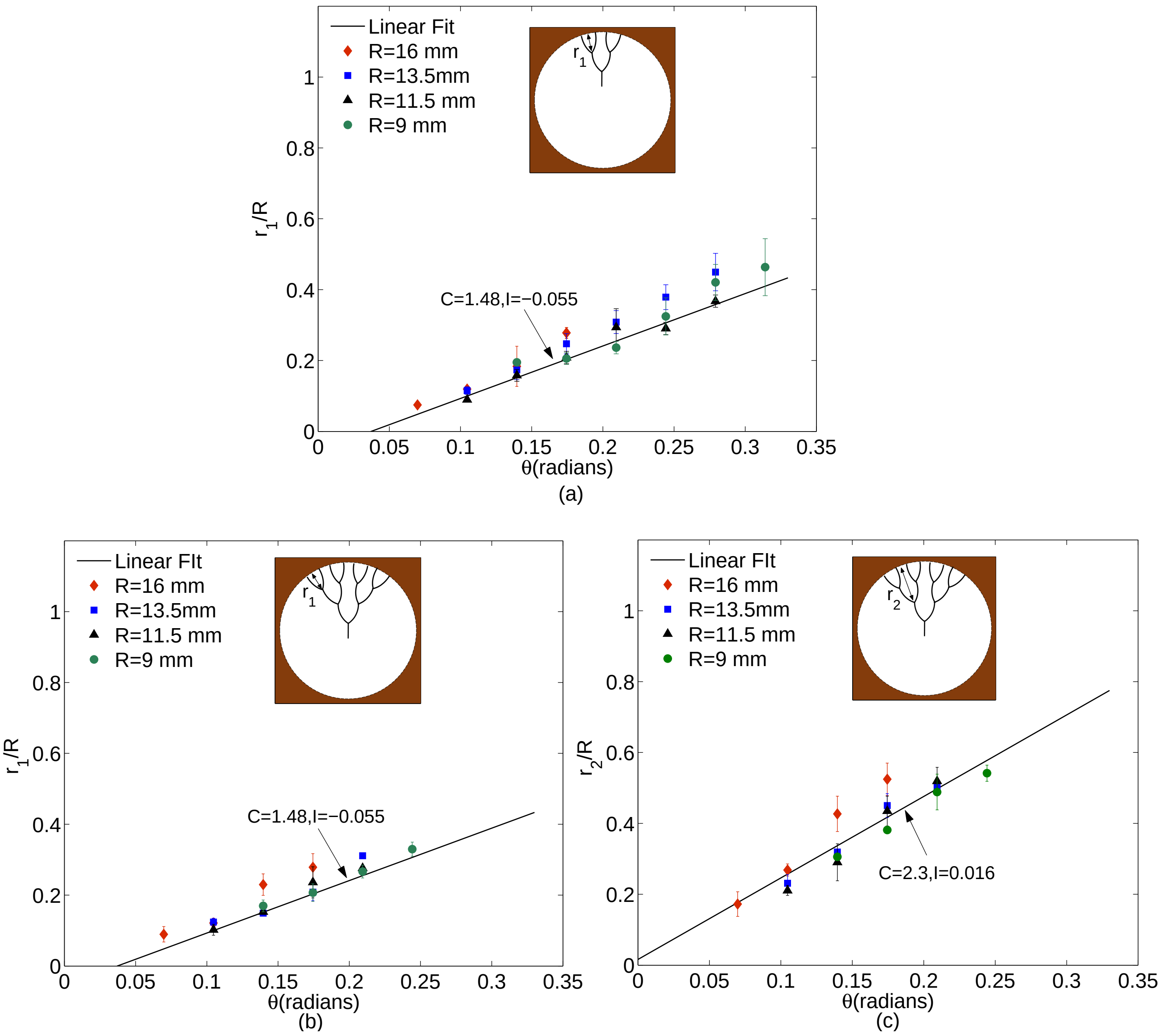

Supplementary Fig. 3. (a) Normalized shielding distances r_1/R in case of structures with coordination number 2. Here again $C = 1.48$ and $I = -0.055$, as is in Fig.3(a) plotted for structure with 1 generation. (b) Normalized shielding distances r_1/R in case of structures with 3 generations. Here again $C = 1.48$ and $I = -0.055$, as is in Fig.3(a) plotted for structure with 1 generation. (c) Normalized shielding distances r_2/R in case of structures with 3 generations. Here again $C = 2.3$ and $I = 0.016$, as is in Fig.3(b) plotted for structure with 2 generations. Data points shown in all cases are for structures fabricated for $R = 9, 11.5, 13.5$, and 16 mm and θ varying from $0 - 0.35$ radians while maintaining $b_0 = 35^{\pm 2} \mu m$.

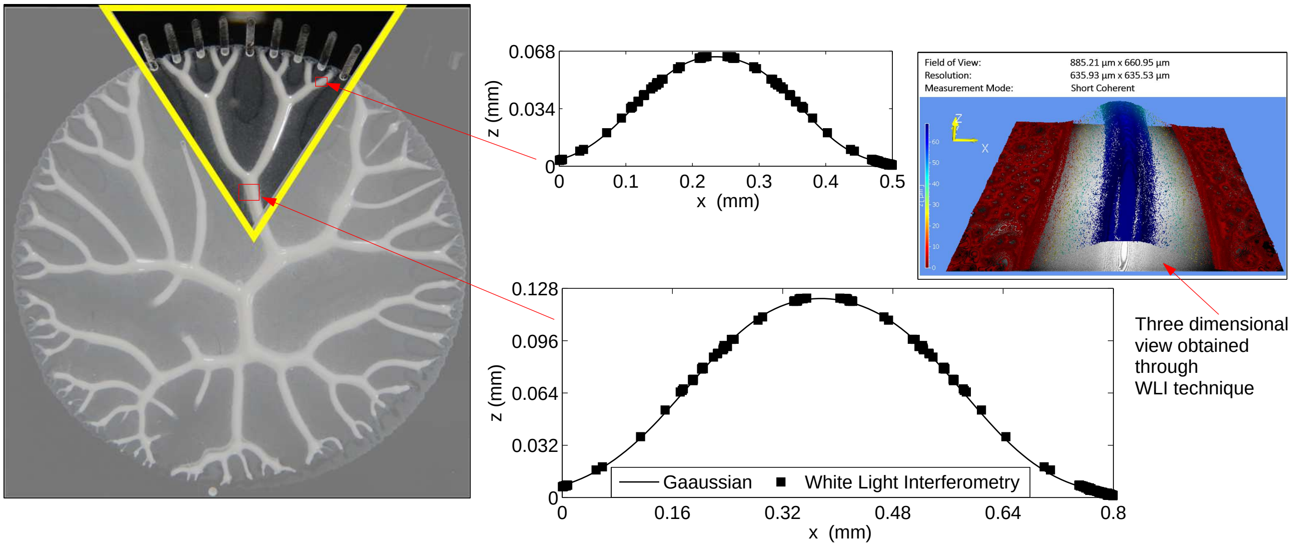

Supplementary Fig. 4. Figure shows the third generation pattern fabricated with $\theta = 0.104$ radians, R=16 mm and $b_0 = 35\mu m$. The three dimensional view of the first generation narrowest branch obtained using white light interferometry technique. The thickness of first, second and third generation in this structure as measure by WLI is 68, 79 and 122 μm respectively. Cross-sectional profile of the first and fourth generation branch is shown as indicated by the arrows

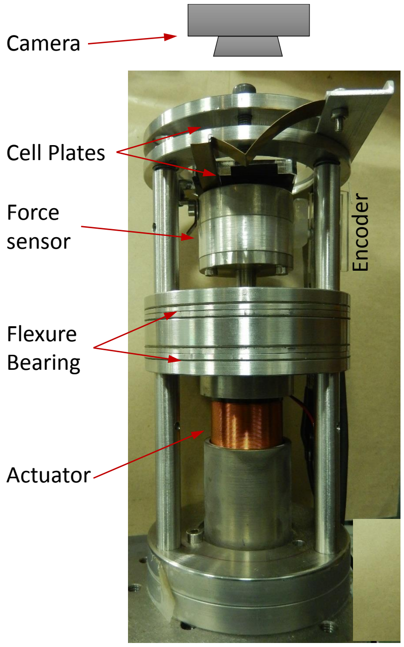

Supplementary Fig. 5. Experimental setup designed, using flexure bearings to operate the cell plates at a very high precision. The different components are actuator, flexure bearings, force sensor, encoder and cell plates. The camera is mounted at the top to record the process as the cell plates are transparent, made from plexi glass.
